# Copper oxide integrated perylene diimide self-assembled graphitic pencil for robust non-enzymatic dopamine detection†

**DOI:** 10.1039/d1ra03908c

**Published:** 2021-07-19

**Authors:** Umay Amara, Sara Riaz, Khalid Mahmood, Naeem Akhtar, Muhammad Nasir, Akhtar Hayat, Muhammad Khalid, Muhammad Yaqub, Mian Hasnain Nawaz

**Affiliations:** Institute of Chemical Sciences, Bahauddin Zakariya University Multan 60800 Pakistan khalidmahmood@bzu.edu.pk; Interdisciplinary Research Centre in Biomedical Materials (IRCBM), COMSATS University Islamabad, Lahore Campus 54000 Pakistan mhnawaz@cuilahore.edu.pk; Department of Chemistry, COMSATS University Islamabad, Lahore Campus 54000 Pakistan; Department of Chemistry, Khwaja Fareed University of Engineering and Technology Rahim Yar Khan 64200 Pakistan

## Abstract

Exploring a robust, extremely sensitive, cost-effective and reliable assay platform for the precise analysis of dopamine (DA) has become a big challenge predominantly at the clinical level. To participate in this quest, herein, we fabricated a perylene diimide (PDI) self-assembled graphitic surface of the graphitic pencil electrode (GPE) anchored copper oxide (CuO). The self-assembled N-rich PDI led to the fast movement of ions by decreasing the bandgap and improved the electron transport kinetics with more exposed catalytic active sites, thus resulting in the robust electrochemical sensing of DA. The designed sensor exhibited good sensitivity (4 μM^−1^ cm^−2^), high structural stability, repeatability and excellent reproducibility with an RSD value of 2.9%. Moreover, the developed system showed a wide linear range (5 μM to 500 μM) and reliable selectivity even in the presence of co-existing interferants, such as ascorbic acid and uric acid. The fabricated nanohybrid was eventually employed to analyze DA in spiked physiological fluids and provided satisfactory recoveries. The designed PDI-CuO based interface also showed a very low detection limit of 6 nM (S/N = 3), consequently confirming its suitability for clinical and biological applications.

## Abstract 2

Exploring a robust, extremely sensitive, cost-effective and reliable assay platform for the precise analysis of dopamine (DA) has become a big challenge predominantly at the clinical level.

## Graphical abstract

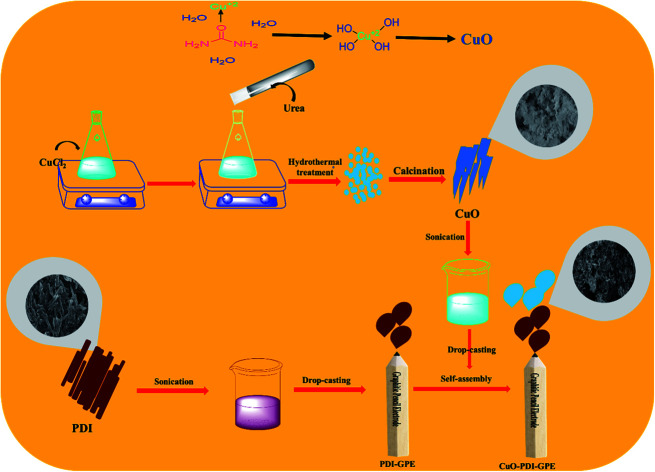

## 1 Introduction

Multiple features of neuronal activity, from neural firing to neuromodulator discharge and signaling, not only trigger brain functions, but also shape animal attitude and behavior.^1^ The current coronavirus pandemic has evoked stress and anxiety, thus resulting in the decreased firing rate of neurotransmitters in general, and for dopamine (DA) in particular.^2–4^ Furthermore, many other diseases, such as senile dementia, Parkinson's disease,^5^ schizophrenia,^6^ epilepsy and HIV infections, have been allied with dysfunction in DA release.^7,8^ Therefore, there is strong demand for the construction of a new generation of analytical tools to report the precise detection and quantification of DA in biological fluids. So far, various analytical techniques, including enzyme-mediated immunosorbent assays, chromatographic, electrochemical and spectroscopic analyses,^9–11^ have been utilized for the detection of DA release levels. However, electrochemical-based measurements have been proved to be the most effective method for both qualitative, as well as quantitative measurements of DA homeostasis. Despite several benefits of achieving good quantitative readouts, these tools fail to provide molecular specificity for the sensitive and selective analysis of DA in the presence of coexisting species.^12^ Therefore, to fabricate an assay platform with good selectivity, high sensitivity, and improved shelf-life with ameliorated electrocatalytic performance is highly desirable.

On the other hands, in recent years, carbon-based organic materials achieved much fame for electrochemical assays due to their biocompatibility and high resistance to fouling.^13^ Moreover, surface defects and the presence of various functional groups in such materials corroborate their sensitivity, owing to the robust electron-transfer. However, this intrinsic charge transfer ability of C-based organic moieties largely depends on the N content;^14^ hence, exploration of C-based materials with excessive nitrogen content bears huge potential. In these lines, the exceptional n-type C-based N-containing semiconductor perylene diimide (PDI) has widely been reported for nano/electrochemical assemblies.^15,16^ PDI provides an easily functionalizable electron-attracting imide fragment with exceptional emissive, chromophoric and redox properties, especially for sensing applications.^17,18^ All these characteristics lead to superior electrical, chemical, thermal and photostability of PDI, along with its exceptional conductivity and sensitivity.^19–22^ However, the ability of PDI molecules to self-aggregate in solution form limits their practical application.^23^

Incorporating a substituent to the imide N of the PDI skeleton *via* self-assembly not only inhibits aggregation, but also preserves the inherent properties of both embodied materials. The nodes in both highest occupied molecular orbital (HOMO) and lowest unoccupied molecular orbital (LUMO) level limit the electronic transitions between the corresponding substituent and PDI nanostructure.^24^ In line with the above discussion, the economically viable copper oxide (CuO) with defined shape and morphology has attracted great interest. Additionally, its unusual remunerations, like high surface area, charge transfer capability and enhanced active sites, lead to high redox activity.^25–27^ Likewise, its ideal stability in a variety of solutions, chemical inertness, availability of plentiful surface oxygen vacancies and biocompatibility make it a potential material for adsorption of positively charged DA.^28,29^ However, inherent shortcomings, like Ostwald ripening, dissolution and aggregation of CuO owing to its high surface energy, lead to its poor electrode stability.^30,31^ The abovementioned problems associated with the hybridization or assembly of CuO make it difficult to get their nanohybrids with greater stability and enhanced electrocatalytic efficacy. Fortunately, CuO has a great tendency to form a stable nanohybrid with electroactive C-based moieties for the integration of a highly stable and electroactive nanocatalyst.^32^ To date, several types of nanohybrids of CuO have been reported with different types of carbon materials, like graphene oxide,^33^ N-doped C quantum dots,^34^ multi-walled-carbon nanotubes,^35^ and polypyrroles.^36^ However, there is still much room to engineer economically worthwhile nanohybrids with ideal stability, sensitivity and lower detection limit.

Herein, we report the synthesis of an economically viable CuO self-assembled PDI nanohybrid with amplified properties without sacrificing the intrinsic properties of both the pristine materials. The composite was drop-casted on the working surface of a graphitic pencil electrode (GPE). The PDI acted as an electron transport mediator that boosted the movement of electrons and ions, owing to the aromatic ring and higher C & N content. Meanwhile, CuO acted as a catalytically active site and facilitated the fast and easy shuttling of electrons between the electroactive interface and analyte, which eventually enhanced the electrocatalytic efficacy for DA sensing.

## 2 Experimental section

### 2.1 Fabrication of CuO nanoparticles

**Scheme 1:**
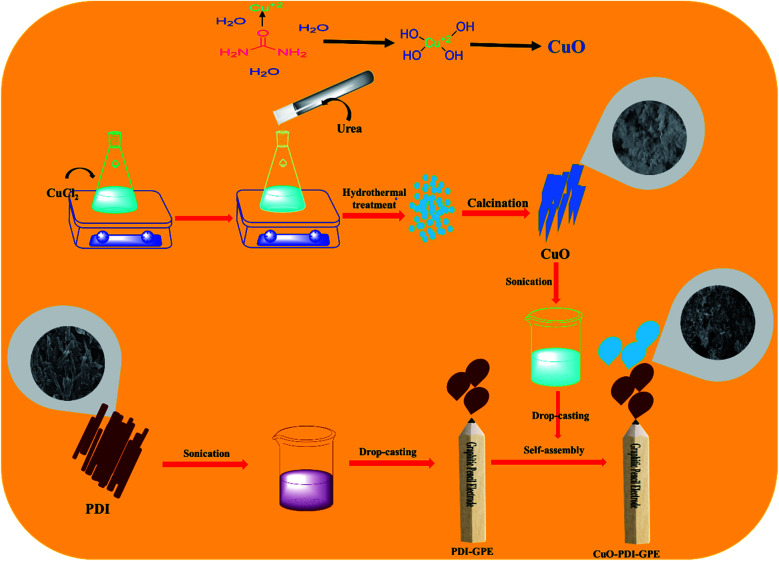
Schematic illustration showing the synthesis and fabrication of the CuO-PDI-GPE based interface.

CuO nanoparticles were synthesized by a simple hydrothermal approach. Briefly, 1 mM solution of CuCl_2_·2H_2_O was prepared in a 50 mL conical flask and kept on stirring to get a clear solution. To this stirred solution, 25 mL of 1 mM urea solution was added dropwise and kept on stirring for 30 minutes. The stirred solution was then transferred to a 100 mL Teflon autoclave, and shifted to the oven for hydrothermal treatment at 180 °C for 8 h. Then, the hydrothermal system was kept at room temperature to cool down. The obtained precipitates were next washed with a mixture of deionized water and ethanol, and kept in the oven overnight at 60 °C for drying. The dried powder was next subjected to calcination for 5 h at 450 °C, which resulted in the formation of CuO nanoparticles,^37^ as shown in Scheme 1.

### 2.2 Self-assembly of PDI-CuO at GPE

Before modification of GPE with perylene diimide, the electrode interface was electrochemically cleaned to oxidize the scums and impurities by continual cyclic voltammetric scans with 0.5 M solution of sulfuric acid (H_2_SO_4_) in the potential bounds of −1.5 to 1.5 V, followed by rinsing with distilled water. To self-assemble perylene-diimide on a working interface, typically, a pencil electrode was placed in 20 μL aqueous solution (3 mg mL^−1^) of PDI for 30 minutes. Furthermore, to electrostatically deposit on the surface of the PDI-modified GPE, the PDI self-assembled GPE was placed in 20 μL (3 mg mL^−1^) of copper oxide solution for 30 minutes. Eventually, the modified electrode surface was dried under ambient conditions, as illustrated from Scheme 1. After drying, the modified GPE was transferred into the electrochemical cell, and tested under different concentrations of DA in PBS with cyclic voltammetry (CV) in the potential range of −0.8 to 0 V. The spikes of DA were added after every 50 s in an electrochemical cell containing 20 mL PBS to analyze the response of the fabricated electrode towards different concentrations of DA. Differential pulse voltammetry (DPV) was performed in the linear bounds of 0–0.4 V with a step size of 25 mV and pulse size of 250 mV. All measurements were performed at a scan rate of 50 mV s^−1^. The GPE (2 mm) was selected as a working interface due to its easy availability, economic viability, and minimal pretreatment requirement. Additionally, the graphitic surface of the sp^2^ hybridized carbon facilitates easy fabrication and good adsorption without any binder.

## 3 Results and discussion

### 3.1 Surface morphology and compositional analysis

**Fig. 1:**
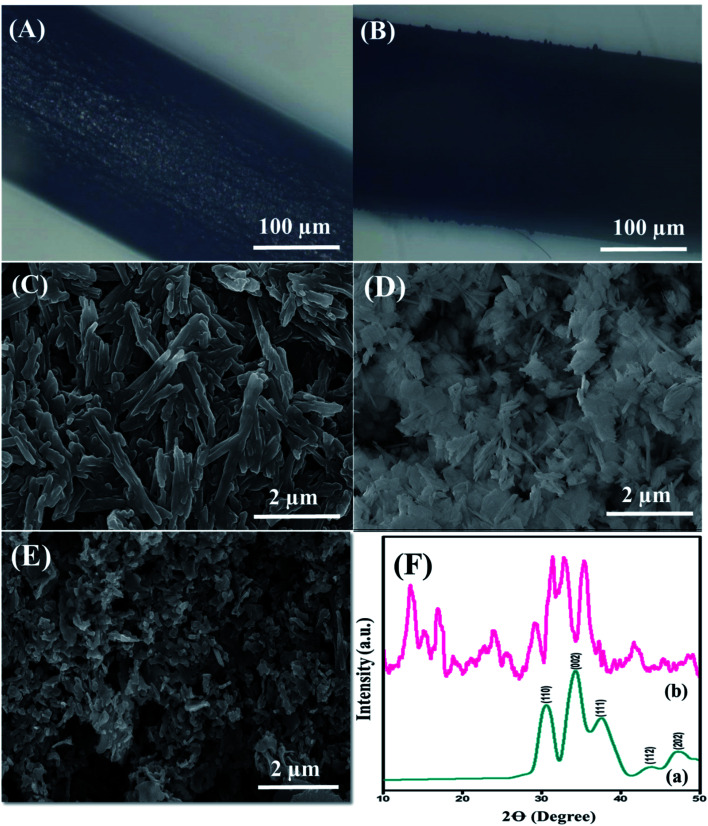
Optical microscopic image of the (A) pristine and (B) CuO-PDI modified graphitic pencil electrode. Scanning electron microscopic images of the (C) PDI, (D) CuO, (E) CuO-PDI modified electrodes. The working interface of GPE was cut, modified and dried at room temperature before microscopic analysis. (F) XRD diffraction of (a) CuO and (b) CuO-PDI.

The surface morphology of the nanohybrid was investigated through electron microscopy and SEM, as depicted in Fig. 1. The displaced microscopic images in Fig. 1(A) and (B) revealed the pristine GPE and uniformly modified GPE interface, respectively. SEM micrographs, as shown in Fig. 1(C), illustrated the equally distributed rod-shaped PDI nanoparticles with an average diameter of 34.5 nm. Meanwhile, Fig. 1(D) represents the homogeneously dispersed CuO nanoparticles with an average diameter of 5 nm. Finally, Fig. 1(E) illustrates plenty of aggregated CuO-embedded PDI molecules with distorted morphology. The distorted shape of the aggregated nanohybrid is due to π–π stacking between PDI and the CuO nanoparticles. This endowed massive active sites for DA absorption that further accelerated the catalytic activity of the integrated surface for DA oxidation. Meanwhile, the EDX analysis in Fig. S3† validates the presence of C, O and Cu from the CuO-enwrapped PDI. The obtained different peak areas were directed in the range of 0–1 keV, 0.5–1 keV and 0.5–1 and 8–10 keV for C, O and Cu, respectively. The phosphorus peak is due to a small amount of impurity.

The crystalline phases of pristine CuO and CuO-enwrapped PDI were analyzed by X-ray diffraction spectroscopy. Pure CuO crystals displayed diffraction peaks of 110, 002, 111, 112, and 202, matching well with (JCPDS 05-0661), as shown in Fig. 1(F)(a).^37^ The diffraction peaks in the range of 15 to 27 validate the presence of carbon owing to PDI, as shown in Fig. 1(F)(b). The results revealed that CuO-PDI has almost the same crystalline phase as that of CuO. A little shift in certain diffraction peaks has been observed due to π–π interactions between both starting materials.^38^ It also validates the incorporation of CuO nanoparticles onto the PDI interface.

**Fig. 2:**
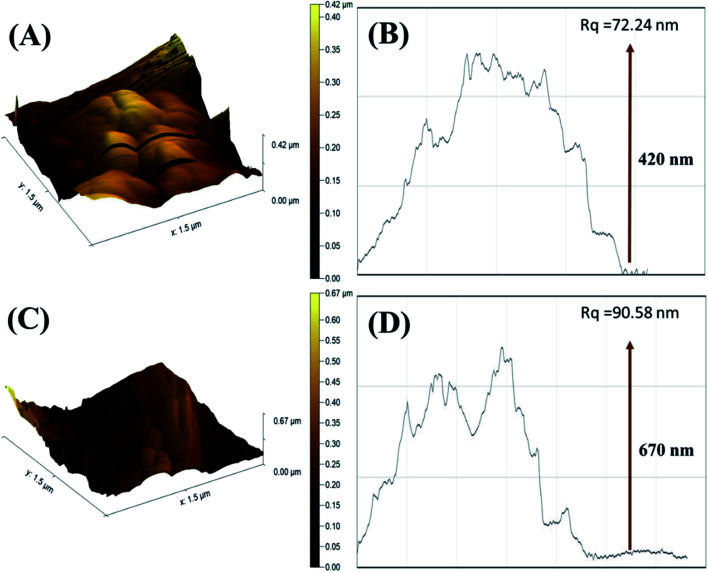
Atomic force microscopy 3D images of (A) PDI; (C) CuO-PDI and their corresponding roughness graphs (B) and (D).

The surface topography of PDI and CuO-PDI was further envisioned *via* AFM topographic analysis. The topological height of the PDI-modified surface was found to be 0.42 μm, as shown in Fig. 2(B). However, after CuO immobilization, the topological height increased to 0.67 μm, as shown in Fig. 2(D). The increase in height is attributed to the homogenous immobilization of metal oxide nanoparticles onto the PDI interface.

The corresponding roughness profile and diagonal root-mean-square roughness (*R*_q_) indicated that the PDI-fabricated interface bears large-sized particles with a roughness of 72.24 nm, as revealed in Fig. 2(A). Meanwhile, the CuO-immobilized PDI interface has equally distributed small-sized particles with increased roughness of 90.58 nm, as shown in Fig. 2(C). It is expected that the uniform distribution of CuO nanoparticles on PDI not only enhanced the topological height, but endowed it with massive active sites that support the maximum dopamine adsorption and enhanced catalytic activity.

### 3.2 Structural analysis and band gaps calculations

**Fig. 3:**
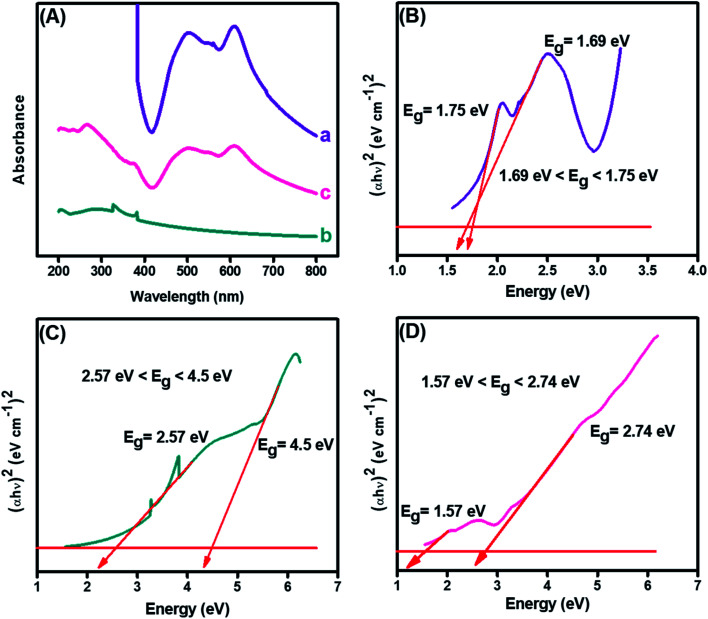
(A) UV-Visible spectrum of (a) PDI, (b) CuO, (c) CuO-PDI, and Tauc plots showing band gap of (B) PDI, (C) CuO, and (D) CuO-PDI.

The structural analysis and formation of the CuO-embedded PDI nanohybrid was initially confirmed from the absorption spectra. Fig. 3(A)(a) illustrated two absorption bands at 501 and 610 nm, which could be credited to the π–π* transitions accompanied by PDI.^39^ A fundamental absorption maximum was shown by CuO, as revealed in Fig. 3(A)(b), at 287 nm that ascribed to the surface plasmon resonance and direct transition of the conduction electrons.^40^ Furthermore, the strong absorption peaks were witnessed at 287, 501 and 510 nm (peaks of both pristine materials), indicating the CuO-PDI nanohybrid formation, owing to π–π interactions between these exemplified materials, as shown in Fig. 3(A)(c).^22,38^

Band gaps of each material was calculated using the Tauc and Davis–Mott relationship that can be extracted from UV data, as shown in eqn (1).^41^

1 (*αhν*)^*n*^ = *K*(*hν* − *E*_g_)

where *hν* represents the photon energy, “*α*” is the molar extinction coefficient, “*k*” represents the energy-independent constant, “*E*_g_” represents the band energy, and “*n*” elucidates the constant exponent, which is 2 in our case for the direct optical transition.

The bandgap of PDI, as shown in Fig. 3(B), was calculated to be 1.75 eV. Meanwhile, the bandgap of the CuO nanoparticles, as depicted from Fig. 3(C), was found to be wider, *i.e.*, up to 4.5 eV. However, the bandgap of CuO (Fig. 3(D)) was decreased after combination with PDI. The bandgap for CuO-PDI was calculated to be 2.74 eV, which also elucidates the formation of a nanohybrid. The redshift in the bandgap energy could be attributed to strong π–π interactions between PDI and the CuO nanoparticles, thus making the developed CuO-PDI nanohybrid stable and an ideal platform for many electrochemical applications.

### 3.3 Functional group and surface defects analysis

**Fig. 4:**
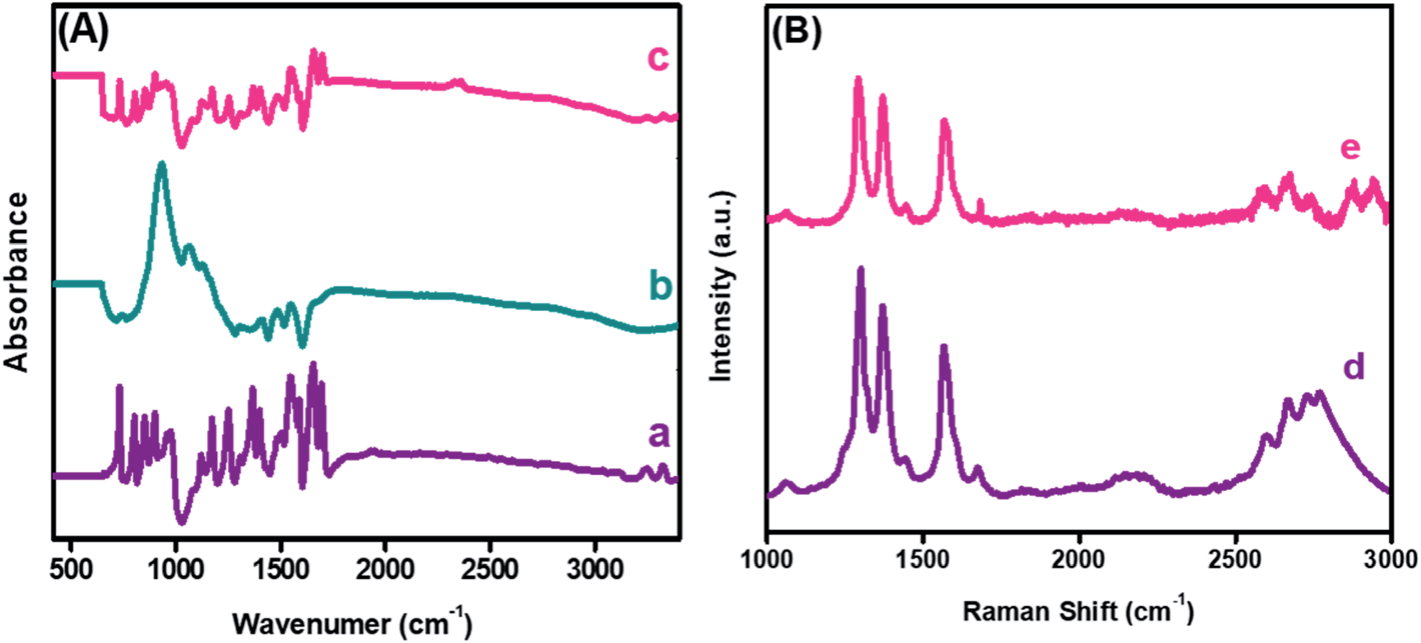
Fourier transform infrared spectroscopy (A) of (a) PDI, (b) CuO, (c) CuO-PDI, and Raman spectroscopy (B) of (d) PDI and (e) CuO-PDI.

The FTIR spectra probed the changes in the chemical functional groups of the embodied materials and nanohybrid. The corresponding spectrum was recorded in absorption mode, as revealed in Fig. 4(A). The absorption bands witnessed at 3242 and 3323 cm^−1^ corresponded to the stretching frequency of the –NH group of PDI, while a sharp band at 1696 cm^−1^ was due to the C=O functionality. Moreover, strong absorption bands at 1656 and 1546 cm^−1^ were due to C=C and C–C of PDI,^42^ as shown in Fig. 4(A)(a). Furthermore, a strong absorption peak was observed at 942 cm^−1^ owing to a characteristic band of the CuO nanoparticles. Likewise, the peaks appearing between 1400 to 1558 cm^−1^ correspond to the symmetrical and asymmetrical stretching frequencies of Cu–O,^43^ as shown in Fig. 4(A)(b). In the case of the nanocomposite, a combination of PDI and CuO bands were observed in Fig. 4(A)(c). A notable reduction in the peak intensities of the –NH and C=O stretching vibration was due to mixed phases and the electrostatic interaction between CuO-PDI. Both UV-Vis and FTIR findings confirmed that CuO interacted with the PDI interface, as illustrated in Scheme 1.

Furthermore, Raman spectroscopy was performed to analyze the sp^2^ hybridized graphitic carbon and defects produced after CuO immobilization. Two distinct bands observed at 1298 and 1369 cm^−1^ correspond to the D and G bands of PDI, as shown in Fig. 4(B)(d). The *I*_D_/*I*_G_ ratio was found to be 1.20. The G band represents the graphitic carbon, and the D band donates the defects introduced by the addition of heteroatoms into a graphitic skeleton. Likewise, a shift in the D band could be envisioned after CuO immobilization, as shown in Fig. 4(B)(e).

The shift corresponds to the successful interaction between the CuO nanoparticles and PDI interface with an *I*_D_/*I*_G_ value of 0.87. This shift offered a smooth pathway for the analyte, and provided the desired selectivity to the integrated interface. This *I*_D_/*I*_G_ ratio also suggested that the metal oxide addition endorsed the maximum structural defects, which enhanced the catalytic activity of the integrated nanohybrid.

### 3.4 Electrocatalytic performance of integrated electrodes

**Fig. 5:**
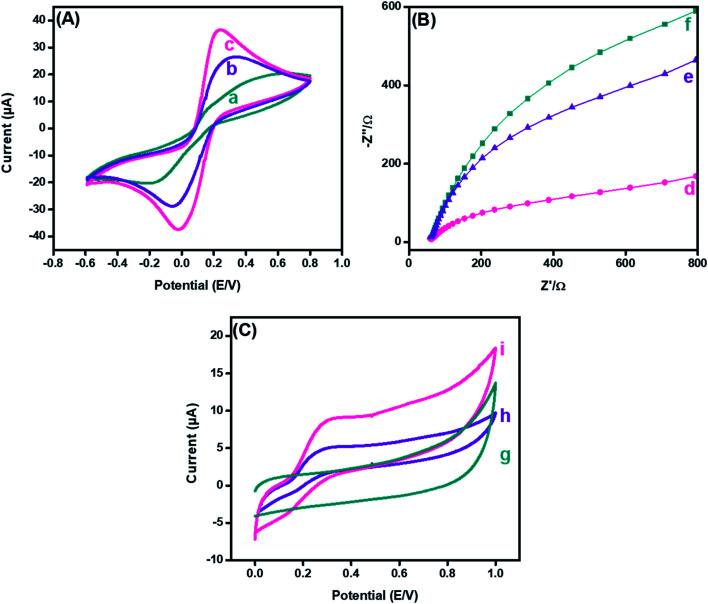
Cyclic voltammetry response (A) of (a) bare, (b) PDI, and (c) CuO-PDI, and electrochemical impedance spectroscopic response (B) of (d) bare, (e) PDI, and (f) CuO-PDI modified GPE in 5 mM [Fe(CN)6]^4−/3−^ (1 : 1). Cyclic voltammetric response (C) of (g) bare, (h) PDI, and (i) CuO-PDI of the modified GPE in 100 μM dopamine in phosphate buffer (pH = 7.4). All measurements were performed at a scan rate of 50 mV s^−1^.

Cyclic voltammetry (CV) has been extensively used to explore electron-transfer initiated chemical reactions during sensor development. The change in the redox peak current and peak-to-peak separation in voltammetric graphs validate the electron transfer at each fabricated step.^44^ The CV findings revealed that the peak-to-peak potential difference (Δ*E* = 0.52 V) of bare GPE decreased to (Δ*E* = 0.36 V) after fabrication with PDI. Additionally, the anodic peak current of the bare electrode (15.57 μA), as revealed in Fig. 5(A)(a), was increased radically (to 23.25 μA) after the deposition of PDI onto the electrode surface (Fig. 5(A)(b)). The increased peak current and decreased peak-to-peak potential difference may refer to the thin film formation, and enhanced electrochemical properties supported by the greater nitrogen content of PDI.^45^ Additionally, the PDI efficacy to generate a stable dianion owing to their strong electron-attracting characteristics contributed towards the amplified current response. However, a great hike in the anodic current (36.4 μA) was observed after the addition of CuO nanoparticles on the PDI-modified GPE, as shown in Fig. 5(A)(c). Moreover, with such increase in the faradic response, a decrease in the peak-to-peak separation (0.24 V) was also perceived compared to the bare and PDI-modified GPE. This ideal response of the nanohybrid fabricated GPE towards the probe is due to PDI that acts as an electron mediator for CuO, which corroborated a greater number of active catalytic sites, resulting in enhanced ion and electron shuttling between the ferro/ferri probe and electrode interface.

The electrochemical active surface area of the integrated nanohybrid fabricated electrode was calculated according to the Randles–Sevcik equation, as follows.^46^

2 *i*_p_ = 2.69 × 10^5^*An*^3/2^*D*^1/2^_red_C* *ν*^1/2^

where “*A*” is the electrochemically active surface area, “*n*” represents a number of electrons involved in the oxidation or reduction of potassium ferro/ferri cyanide, which in the present case is 1. “C*” represents the concentration of ferrocyanide (5 × 10^−3^ M), “*D*_red_” elucidates the diffusion coefficient of potassium ferrocyanide (7.6 × 10^−6^ cm^2^ s^−1^) and “ν” elucidates the scan rate (50 × 10^−3^ V). The electrochemically active surface area (EASA) of CuO-PDI was calculated to be 0.09 cm^2^. Such a large active surface area offered robust tunneling of an electron across the interphase, and eventually resulted in enhanced electrocatalytic performance.

A similar phenomenon for the electron-transfer resistance was observed by electrochemical impedance spectroscopy to authorize the electrochemical functioning of the fabricated electrode. The typical Nyquist plot of the impedance spectra, Fig. 5(B) demonstrated the electron transfer that is correspondent from a semicircle (at high frequencies) and diffusion-limited processes from linear portions (at low frequencies).^47^ The resistance of the bare electrode, as shown in Fig. 5(B)(d), was reasonably declined after PDI deposition, as shown in Fig. 5(B)(e). The minimized resistance was attributed to the thin film formation of PDI on the electrode surface with improved surface area owing to the stable dianion formation. Likewise, the electron-deficient imide moiety promoted the electron transference from the redox probe to the electrode interface. In addition, it was observed from the EIS response that the addition of CuO onto PDI caused a further decrease in the resistance of the transducing interface, as shown in Fig. 5(B)(f). These results recommended that the efficacy of the fast electron transmission of the CuO-PDI fabricated electrode is due to contact of a greater number of active sites compared to the pristine and PDI-modified GPE. This prominent improvement in the impedimetric response after modification paves the way towards establishment of an improved sensing surface.

Furthermore, CV was utilized to analyze the electrochemical behavior of bare, PDI and CuO-PDI towards 100 μM DA at a scan rate of 50 mV s^−1^, as shown in Fig. 5(C). The bare electrode showed no response towards DA, as shown in Fig. 5(C)(g). However, reversible redox peaks were witnessed at the interface of the PDI and CuO-PDI fabricated electrodes. The PDI fabricated electrodes showed a lower oxidation current (4.9 μA), as revealed in Fig. 5(C)(h), compared to the CuO-PDI modified GPE (8.8 μA), as shown in Fig. 5(C)(i). This 2-fold hike in the current could be accredited to the good electronic communication between PDI and catalytic CuO, which led to a stable interface with an amplified faradic response. Meanwhile, the electroactive behavior of CuO, along with a high specific surface area and oxygen moieties of CuO promoted enhanced dopamine coverage at the catalytic interface.^48,49^ Such improved interaction between the catalytic surface and analyte facilitated the electron transfer kinetics and enhanced electrocatalytic efficacy of the fabricated catalytic platform for DA oxidation.

### 3.5 Reaction kinetics and mechanistic elucidation at the integrated CuO-PDI-GPE interface

To examine the surface activity and reaction kinetics at the surface of the synthesized nanohybrid, cyclic voltammetry was performed in the presence of 70 μM of DA with the scan rates ranging from 50 mV s^−1^ to 325 mV s^−1^.

**Fig. 6:**
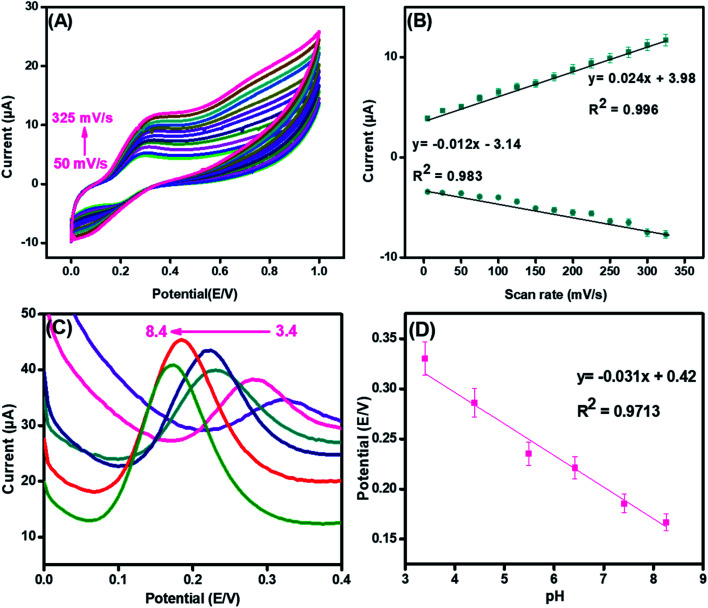
Cyclic voltammetric response (A) and its corresponding linear graph (B) of CuO-PDI-GPE in the presence of 70 μM DA in phosphate buffer (pH 7.4) at scan rates from (50 mV s^−1^ to 325 mV s^−1^). Differential pulse voltammetric response (C) and its corresponding linear graph (D) of CuO-PDI-GPE in the presence of 100 μM DA in phosphate buffer at a scan rate of 50 mV s^−1^ in pH 3.4 to 8.4.

In Fig. 6(A), both oxidation and reduction peaks are present. The result demonstrated that both oxidation and reduction peaks increased linearly with a scan rate; thus, a redox reaction is taking place at the integrated CuO-PDI-GPE interface. This phenomenon is consistent with the characteristic irreversible reaction.^50,51^ Meanwhile, the much larger anodic peak current (in contrast to the cathodic current) also corroborated the electrochemical irreversibility of the reaction.^52^ Moreover, upon successive current amplification with the increase in scan rate, a slight potential shift in the CV response was also observed, which indicated the increased absorption or surface coverage of DA at the electrode surface.^17,53–55^ The calibration plot of the scanning speed against the redox peak currents revealed a characteristic adsorption-controlled process, as shown in Fig. 6(B).^56^

3 *y* = 0.02*ν* mV s^−1^ + 3.98 (*R*^2^ = 0.996) 

Moreover, a linear relationship was observed between the square root of the scan rate and the anodic peak current, as shown in Fig. S1(A),† validating an adsorption controlled mechanism.^57^

4 *I*_P_ = 056*ν* mV s^−1^ + 0.855 (*R*^2^ = 0.983)

Meanwhile, a linear regression equation was obtained by plotting a graph between the natural log of the scan rates and anodic peak currents, as revealed in Fig. S1(b).†

5 *ln* *I*_P_= 0.44*ν* mV s^−1^ − 0.154 (*R*^2^ = 0.981)

In addition, a linear relation was developed when a graph was plotted between the natural log of the scan rate and peak potential (*E*_p_), as shown in Fig. S1(C).†

6 *E*_p_ = 0.034 *ln* *ν* + 0.14 (*R*^2^ = 0.984)

Based on these findings, the charge transfer coefficient *α* and standard rate constant for *K*_s_ between the integrated interface and surface-confined redox couple was premeditated from Laviron's equation.^58^7
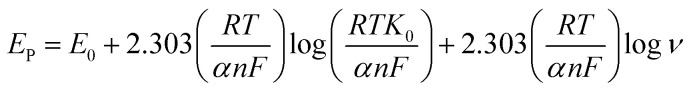
where *R*, *T*, *α*, and *F* represent the anodic, formal potential of the integrated electrode system, general gas constant, absolute temperature, electron transfer coefficient, and Faraday constant, respectively, and *n* represents the number of electrons transferred. The value of *αn* is calculated from the slope in Fig. S1(C).† Using these plots at pH 7.4, *α* is calculated to be 0.86 for the adsorption-controlled reaction. Using eqn (7), the number of electrons transferred at the interface of CuO-PDI-FPE was found to be 2.01, thus suggesting the two-electron transfer steps coupled to two protons mechanism.^59^

**Scheme 2:**
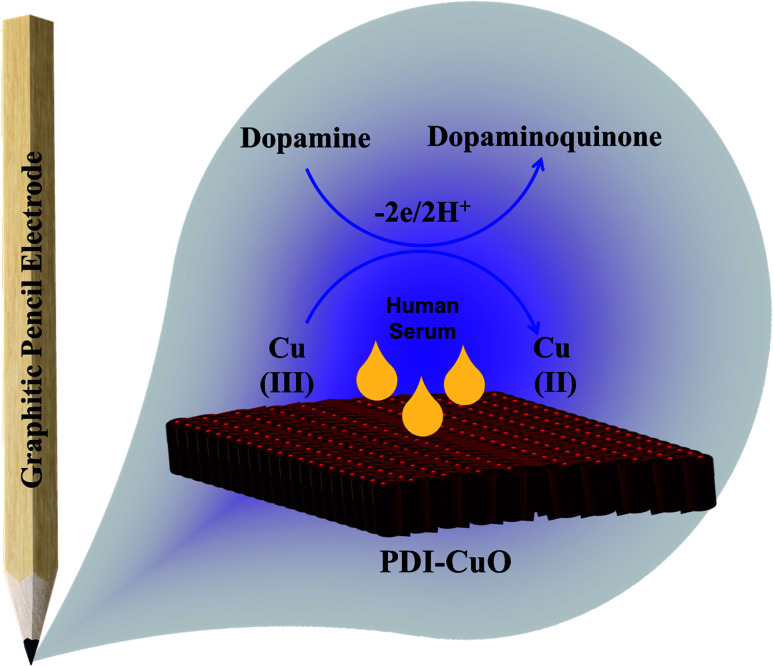
Schematic design showing the electrooxidation of DA at the PDI-CuO-GPE interface.

Furthermore, it can be inferred that the increase in peak current, as revealed previously in Fig. 5(C) during the electrochemical oxidation of DA, is due to the release of two electrons and proton into the conduction band of the sensor substrate, as illustrated in Scheme 2 and eqn (7). Moreover, the surface of the integrated CuO-PDI-GPE is highly electroactive due to the presence of CuOOH that acts as a catalytic center with fast shuttling capability. Besides this, the surface is also endowed with a high content of N and C due to the presence of PDI that boosts the movement of ions by performing as an electron transport booster or mediator. Thus, the catalytically active layer of CuOOH upon interaction with DA causes a robust and smooth oxidation to dopamine-*o*-quinone and itself reduces to Cu(OH)_2_ with (2+),^37,60^ as shown in Scheme 2.

Briefly, this process involves two electrons and protons to form the final oxidation product, *i.e.*, dopamine-*o*-quinone.^61^8
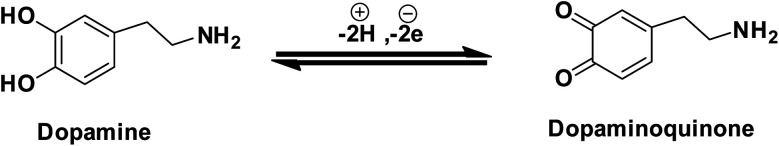


Likewise, the pH of the electrolyte has a great influence on electrochemical analysis, as it alters both Faradaic current and potential. Fig. 6(C) illustrates the effect on the electrolytic oxidation potential/current of DA (100 μM) at the CuO-PDI-GPE interface by DPV in PBS of pH from 3.4 to 8.4. The results revealed an increase in the Faradaic current up to pH 7.4; whereas, it started decreasing upon further increase of pH up to 8.4. On the other side, the oxidation potential shifted towards a lower value upon increasing the pH up to 8.4.^62^ These findings suggested that pH 7.4 delivered an appropriate redox potential and current for DA. Thus, pH 7.4 was selected as the optimal pH for all experiments. A good linear correlation among the pH and redox potential was achieved with the regression equation, as shown in Fig. 6(D). This linear plot suggested the involvement of the same electron and proton numbers in the electro-oxidation of DA at CuO-PDI-GPE, thus corroborating the previous results.

Furthermore, we calculated the electron transfer rate constant, exploiting Laviron eqn (9). The *K*_s_ for DA came out to be 0.76 s^−1^.^57^9
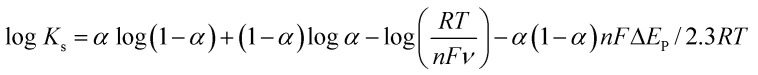


### 3.6 Sensing efficacy of the integrated CuO-PDI-GPE electrode system

**Fig. 7:**
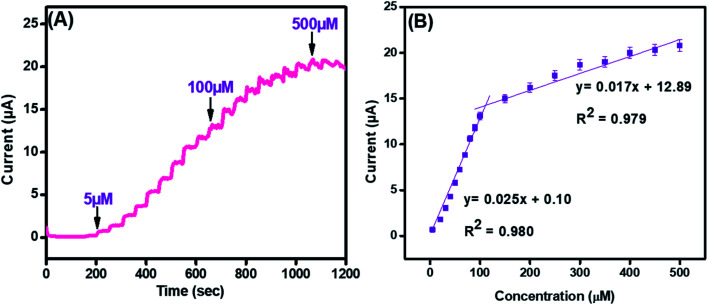
Amperometry (A) and its corresponding linear graph (B) for CuO-PDI-GPE at concentrations ranging from 5 μM to 500 μM in phosphate buffer (pH 7.4) at a scan rate of 50 mV s^−1^.

Amperometry was employed to find the sensing efficacy of the integrated CuO-PDI-GPE based catalytic interface in the minimum time with the maximum faradic response in the linear bounds of 5 μM to 500 μM at an applied potential of (0.57 V), as shown in Fig. 7(A). Results also indicated an increase in the amperometric current response with a fast response time of (∼3 s). The subsequent calibration plot (Fig. 7(B)) of the modified electrode also exhibited an exceptionally linear response between the current and DA concentration. It demonstrated that the electrocatalytic behavior of the nanohybrid fabricated electrode is highly concentration-dependent for DA sensing. Besides amperometry, current-sensitive DPV was also performed to endorse our findings, as revealed in Fig. S2(A and B).† The response of the integrated catalytic electrode material was just the same, as perceived in the case of amperometry. Besides the successive current amplification with increase in DA concentration, a slight potential shift in DPV was also observed, which indicates the increased absorption or surface coverage of dopamine at the electrode surface.^53–55^

The standard method was employed to calculate the limit of detection, which was premeditated to be 6 nM (S/N = 3). This very low detection limit accentuated that the developed catalytic interface is suitable for its practical application in biological serum for dopamine detection. Similarly, the sensitivity was found to be (4 μA μM^−1^ cm^−2^). These outcomes are in great competition with the previously reported literature, as shown in Table S1.† Moreover, the surface coverage (*Γ*) of dopamine on CuO-PDI-GPE can also be calculated by using the Laviron eqn (10),10
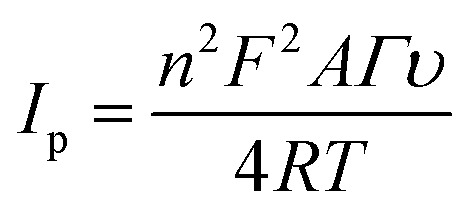
where “*n*” signifies the number of electrons transferred, which was found to be 2 in this case, “*F*” represents the Faraday constant, “*A*” represents the area of the graphitic pencil electrode, and “*R*” and “*T*” represent the general constants. The surface coverage of DA on the working electrode surface was calculated to be 6.2 × 10^−4^ mol cm^−2^. The higher value of surface coverage elucidated the increased electron transfer kinetics and enhanced ability of the sensor towards dopamine detection.

### 3.7 Selectivity, repeatability, reproducibility and stability of the integrated CuO-PDI-GPE electrode system

**Fig. 8:**
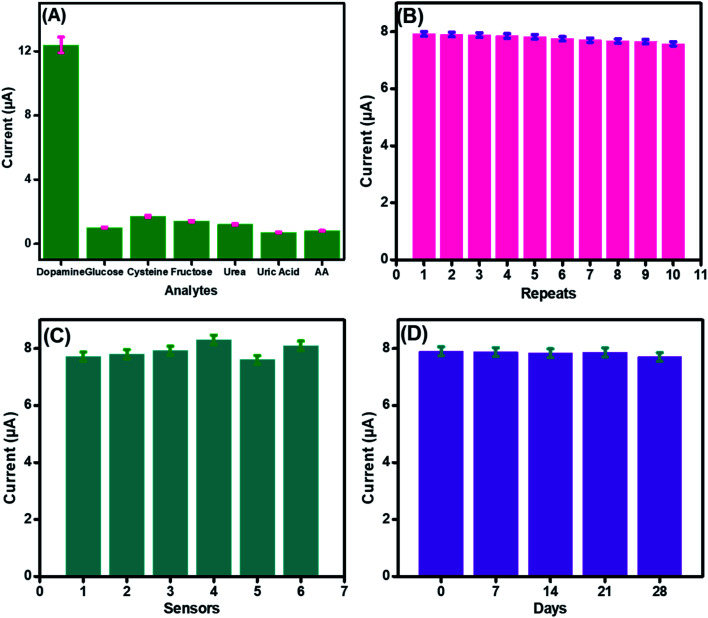
Current response (A) of CuO-PDI-GPE in the presence of 100 μM DA and 1000 μM interfering species in phosphate buffer (pH 7.4) at a scan rate of 50 mV s^−1^. Repeatability (B) data of the CuO-PDI-GPE fabricated electrodes for their ten times response towards dopamine. Reproducibility (C) of the six CuO-PDI-GPE fabricated electrodes for their response towards dopamine under a similar environment. Stability (D) of the CuO-PDI-GPE fabricated electrode and its response towards dopamine for 28 days with analysis intervals of 7 days each.

To authenticate the proficient working of the fabricated sensor in a complex biological environment and to inspect its specificity in the presence of possible interfering species, electrochemical detection of DA was performed in the presence of possible interferants, as shown in Fig. 8(A). A sudden increase in the current was observed after dopamine addition. However, a very small increase in the amperometric current signals was observed after the spiking of a 10-fold excess of interfering species, like cysteine, glucose, fructose, and urea, owing to the higher electronegativity of oxygen accompanied by CuO that inhibits complexation with these interfering analytes.^63^

The performance of the developed CuO-PDI nanohybrid-based sensor was not even affected much by uric acid and ascorbic acid. This might be attributed to the fact that uric acid and ascorbic acid have negative charges at pH 7.4, while DA has a positive charge. Furthermore, the presence of negatively charged free hydroxyl groups on the surface of CuO led to electrostatic interaction with the positively charged DA, while a strong repulsion was produced by the electrode surface for the negatively charged interfering species.^64^

The repeatability, reproducibility, and stability of the fabricated CuO-PDI-GPE were also analyzed and verified under comparable parameters. The standard deviation was found to be 1.4% after repeated use of the same electrode (*n* = 10), demonstrating excellent repeatability of the prepared sensor, as shown in Fig. 8(B). In order to estimate the reproducibility of the primed sensor, 6 electrodes were analyzed for DA detection under similar experimental conditions, as graphed in Fig. 8(C). The standard deviation, in this case, was found to be 2.9%, authorizing the good reproducibility of the fabricated sensor. Likewise, the storage stability of the proposed sensor was also assessed. The prepared sensor was stored under ambient conditions and was tested against DA after every 7 days, as revealed in Fig. 8(D). The relative standard deviation was calculated to be 1.2%, approving the long-term stability of the proposed sensor.^65^ The excellent repeatability, reproducibility, and stability were due to the stable catalytic interface.

### 3.8 Real-time application of integrated CuO-PDI-GPE

The foremost challenge in the sensor field is to develop sensors with the capability to work selectively in the complex biological environment, and making them applicable for clinical diagnostics. Hence, the fabricated sensor was also evaluated for real-life application in human serum samples under optimized test conditions. Human serum samples were prepared by diluting it 50 times with PBS before analysis, followed by spiking the known concentrations of DA. The outcomes are displayed in Table 1, indicating minor fluctuations in the values of recoveries ranging from 99.97 to 100.10%. The satisfactory output in human fluids proved that CuO-PDI-GPE can be a competent nanohybrid for clinical diagnostics.

**Table 1:** The recovery data of the CuO-PDI-GPE in human blood serum for dopamine detection (*n* = 3)

Sample No.	Added (μM)	Found (μM)	Relative standard deviation (%)	Relative error (%)	Recovery (%)
1	100	97	0.43	0.03	99.97
2	250	220	0.45	0.12	100.10
3	500	450	0.17	0.10	100.10

Moreover, the outcomes of the developed DA sensor were compared with formerly reported sensors, as shown in Table S1.† The integrated nanohybrid showed great performance compared to previously reported data. The good sensitivity, stability, lower detection limit, wider linear range, and a larger value of DA coverage on the electrode interface could be attributed to the synergistic effects of both embodied materials.

## 4 Conclusion

We reported a simple non-enzymatic electrochemical sensor for DA based on the PDI-immobilized CuO nanoparticles-coated graphitic pencil electrode. Uniform immobilization was achieved through negatively charged nitrogen atoms present on PDI. The immobilization of PDI on CuO leads to the fast kinetics of the ions with a low bandgap, thus resulting in enhanced electrocatalytic efficacy. Additionally, the synergistic effect of CuO with N-rich PDI leads to the exposition of more catalytic active sites, thus resulting in fast electrocatalytic efficacy towards DA monitoring. The sensor also shows good linear range, high stability, presentable repeatability, and reproducibility with reliable selectivity. The developed sensor successfully monitored DA from human serum samples; thus, it holds great potential for its further exploitation towards diagnostic applications in real life.

## Conflicts of interest

There are no conflicts to declare.

## Supplementary Material

RA-011-D1RA03908C-s001

## References

[cit1] Patriarchi T., Cho J. R., Merten K., Marley A., Broussard G. J., Liang R., Williams J., Nimmerjahn A., von Zastrow M., Gradinaru V. (2019). Nat. Protoc..

[cit2] Numa C., Nagai H., Taniguchi M., Nagai M., Shinohara R., Furuyashiki T. (2019). Sci. Rep..

[cit3] Lynch A., Bastiampillai T., Dhillon R. (2021). Australian & New Zealand Journal of Psychiatry.

[cit4] Li J., Li X., Jiang J., Xu X., Wu J., Xu Y., Lin X., Hall J., Xu H., Xu J. (2020). Front. Psychiatr..

[cit5] Poewe W., Seppi K., Tanner C. M., Halliday G. M., Brundin P., Volkmann J., Schrag A.-E., Lang A. E. (2017). Nat. Rev. Dis. Primers.

[cit6] Arnsten A. F., Girgis R. R., Gray D. L., Mailman R. B. (2017). Biol. Psychiatr..

[cit7] Redgrave P., Gurney K. (2006). Nat. Rev. Neurosci..

[cit8] Merims D., Giladi N. (2008). Park. Relat. Disord..

[cit9] Gu H., Varner E. L., Groskreutz S. R., Michael A. C., Weber S. G. (2015). Anal. Chem..

[cit10] Zhao J., Zhao L., Lan C., Zhao S. (2016). Sensor. Actuator. B Chem..

[cit11] Zhu L., Xu G., Song Q., Tang T., Wang X., Wei F., Hu Q. (2016). Sensor. Actuator. B Chem..

[cit12] Numan A., Shahid M. M., Omar F. S., Ramesh K., Ramesh S. (2017). Sensor. Actuator. B Chem..

[cit13] Cuniberto E., Alharbi A., Wu T., Huang Z., Sardashti K., You K.-D., Kisslinger K., Taniguchi T., Watanabe K., Kiani R. (2020). Sci. Rep..

[cit14] Hayat K., Munawar A., Zulfiqar A., Akhtar M. H., Ahmad H. B., Shafiq Z., Akram M., Saleemi A. S., Akhtar N. (2020). ACS Appl. Mater. Interfaces.

[cit15] Khan Q. U., Tian G., Bao L., Qi S., Wu D. (2018). New J. Chem..

[cit16] Amara U., Mahmood K., Riaz S., Nasir M., Hayat A., Hanif M., Yaqub M., Han D., Niu L., Nawaz M. H. (2021). Microchem. J..

[cit17] Amara U., Mehran M. T., Sarfaraz B., Mahmood K., Hayat A., Nasir M., Riaz S., Nawaz M. H. (2021). Microchim. Acta.

[cit18] Huang C., Barlow S., Marder S. R. (2011). J. Org. Chem..

[cit19] Bu L., Dawson T. J., Hayward R. C. (2015). ACS Nano.

[cit20] Li S., Zhao C., Zhou S., Zhang Y., Zhu P., Yu J. (2019). Chem. Eng. J..

[cit21] Ma Z., Fu H., Meng D., Jiang W., Sun Y., Wang Z. (2018). Chem. Asian J..

[cit22] Muthuraj B., Mukherjee S., Chowdhury S. R., Patra C. R., Iyer P. K. (2017). Biosens. Bioelectron..

[cit23] Barendt T. A., Ferreira L., Marques I., Félix V., Beer P. D. (2017). J. Am. Chem. Soc..

[cit24] Zhang F., Ma Y., Chi Y., Yu H., Li Y., Jiang T., Wei X., Shi J. (2018). Sci. Rep..

[cit25] Liu B., Ouyang X., Ding Y., Luo L., Xu D., Ning Y. (2016). Talanta.

[cit26] Soomro R. A., Hallam K. R., Ibupoto Z. H., Tahira A., Jawaid S., Sherazi S. T. H., Willander M. (2015). RSC Adv..

[cit27] Verma N., Kumar N. (2019). ACS Biomater. Sci. Eng..

[cit28] Rajamani A. R., Peter S. C. (2018). ACS Appl. Nano Mater..

[cit29] Balasubramanian P., Balamurugan T., Chen S.-M., Chen T.-W., Sathesh T. (2018). ACS Sustainable Chem. Eng..

[cit30] Zhang L., Wang H. (2011). ACS Nano.

[cit31] Dörner L., Cancellieri C., Rheingans B., Walter M., Kägi R., Schmutz P., Kovalenko M. V., Jeurgens L. P. (2019). Sci. Rep..

[cit32] Song Z., Liu W., Sun N., Wei W., Zhang Z., Liu H., Liu G., Zhao Z. (2019). Solid State Commun..

[cit33] Kumar S. K., Mamatha G., Muralidhara H., Anantha M., Yallappa S., Hungund B., Kumar K. Y. (2017). J. Sci.: Adv. Mater. Devices.

[cit34] Muthusankar G., Sethupathi M., Chen S.-M., Devi R. K., Vinoth R., Gopu G., Anandhan N., Sengottuvelan N. (2019). Compos. B Eng..

[cit35] Zhou X., Nie H., Yao Z., Dong Y., Yang Z., Huang S. (2012). Sensor. Actuator. B Chem..

[cit36] Yu H., Jian X., Jin J., Zheng X.-c., Liu R.-t., Qi G.-c. (2015). Microchim. Acta.

[cit37] Asad M., Zulfiqar A., Raza R., Yang M., Hayat A., Akhtar N. (2020). Electroanalysis.

[cit38] Chen M., Ding Y., Gao Y., Zhu X., Wang P., Shi Z., Liu Q. (2017). RSC Adv..

[cit39] Hariharan P., Pitchaimani J., Madhu V., Anthony S. P. (2017). Opt. Mater..

[cit40] Dhineshbabu N., Rajendran V., Nithyavathy N., Vetumperumal R. (2016). Appl. Nanosci..

[cit41] Suram S. K., Newhouse P. F., Gregoire J. M. (2016). ACS Comb. Sci..

[cit42] Mattson M. A., Green T. D., Lake P. T., McCullagh M., Krummel A. T. (2018). J. Phys. Chem. B.

[cit43] Huang B.-R., Wang M.-J., Kathiravan D., Kurniawan A., Zhang H.-H., Yang W.-L. (2018). ACS Appl. Bio Mater..

[cit44] Nawaz M. H., Hayat A., Catanante G., Latif U., Marty J. L. (2018). Anal. Chim. Acta.

[cit45] Ören T., Birel Ö., Anık Ü. (2018). Anal. Lett..

[cit46] Tigari G., Manjunatha J. (2019). J. Sci.: Adv. Mater. Devices.

[cit47] Lee S.-K., Song M.-J., Kim J.-H., Kan T.-S., Lim Y.-K., Ahn J.-P., Lim D.-S. (2014). NPG Asia Mater..

[cit48] Zhang F., Li Y., Gu Y.-e., Wang Z., Wang C. (2011). Microchim. Acta.

[cit49] Huang Y., Tan Y., Feng C., Wang S., Wu H., Zhang G. (2019). Microchim. Acta.

[cit50] Li J., Jiang J., Xu Z., Liu M., Feng H., Liu Y., Qian D. (2017). Microchim. Acta.

[cit51] Ning J., He Q., Luo X., Wang M., Liu D., Wang J., Liu J., Li G. (2018). Sensors.

[cit52] Hsu M.-S., Chen Y.-L., Lee C.-Y., Chiu H.-T. (2012). ACS Appl. Mater. Interfaces.

[cit53] Hobbs C. N., Johnson J. A., Verber M. D., Wightman R. M. (2017). Analyst.

[cit54] Reddy S., Swamy B. K., Jayadevappa H. (2012). Electrochim. Acta.

[cit55] Mphuthi N. G., Adekunle A. S., Fayemi O. E., Olasunkanmi L. O., Ebenso E. E. (2017). Sci. Rep..

[cit56] Yan X., Gu Y., Li C., Tang L., Zheng B., Li Y., Zhang Z., Yang M. (2016). Biosens. Bioelectron..

[cit57] Zhou Y., Tang W., Wang J., Zhang G., Chai S., Zhang L., Liu T. (2014). Anal. Methods.

[cit58] Nasirizadeh N., Shekari Z., Zare H. R., Ardakani S. A., Ahmar H. (2013). J. Braz. Chem. Soc..

[cit59] Kokulnathan T., Anthuvan A. J., Chen S.-M., Chinnuswamy V., Kadirvelu K. (2018). Inorg. Chem. Front..

[cit60] Sundar S., Venkatachalam G., Kwon S. J. (2018). Nanomaterials.

[cit61] Segura-Aguilar J. and Paris I., Handbook of Neurotoxicity, ed, Kostrzewa RM, Springer, New York, 2014, pp. 865–883

[cit62] Zhang X., Ma L.-X., Zhang Y.-C. (2015). Electrochim. Acta.

[cit63] Jiang L.-C., Zhang W.-D. (2010). Biosens. Bioelectron..

[cit64] Jiang G., Gu X., Jiang G., Chen T., Zhan W., Tian S. (2015). Sensor. Actuator. B Chem..

[cit65] Yuan D., Chen S., Yuan R., Zhang J., Liu X. (2014). Sensor. Actuator. B Chem..

