# Supplementary material for: Copper oxide integrated perylene diimide self-assembled graphitic pencil for robust non-enzymatic dopamine detection
Source: RSC Adv. 2021 Jul 19;11(40):25084–95. doi: 10.1039/d1ra03908c (PMC9036951; doi:10.1039/d1ra03908c)
Supplement: RA-011-D1RA03908C-s001 [file RA-011-D1RA03908C-s001.pdf]

Electronic Supplementary Material (ESI) for RSC Advances.
This journal is © The Royal Society of Chemistry 2021

SUPPORTING INFORMATION

Copper Oxide Integrated Perylene Diimide Self-assembled Graphitic Pencil For Robust Non-Enzymatic Dopamine Detection

Umay Amara,[1,2] Sara Riaz,[3] Khalid Mahmood,[*1] Naeem Akhtar,[2] Muhammad Nasir,[2] Akhtar Hayat,[2] Muhammad Khalid,[4] Muhammad Yaqub,[1] Mian Hasnain Nawaz[*2]

[1] *Institute of Chemical Sciences, Bahauddin Zakariya University, Multan 60800, Pakistan*

[2] *Interdisciplinary Research Centre in Biomedical Materials (IRCBM), COMSATS University Islamabad, Lahore Campus 54000, Pakistan*

[3] *Department of Chemistry, COMSATS University Islamabad, Lahore Campus 54000, Pakistan*

[4]*Department of Chemistry, Khwaja Fareed University of Engineering and Technology, Rahim Yar Khan, 64200, Pakistan*

Correspondence: (M.H.N.*) mhnawaz@cuilahore.edu.pk; khalidmahmood@bzu.edu.pk

1 Experimental Section

1.1 Chemicals

PDI, $CuCl_2.2H_2O$, urea, potassium ferrocyanide ($K_4Fe(CN)_6$), phosphate Buffer Saline (PBS), potassium ferricyanide ($K_3Fe(CN)_6$), glucose, fructose, ascorbic acid, urea, uric acid and, cysteine were bought from Sigma-Aldrich and used as received. Human serum samples were collected from the lab of a local hospital on voluntarily basis and stored at 4°C. All aqueous solutions were prepared with doubly distilled water.

UV-Vis spectrums were observed on Perkin Elmer Lambda 25 UV-Vis spectrometer within the bounds of 800-200 nm. FTIR spectroscopy was conducted on Thermo Scientific Nicolet 6700 in ATR mode. To analyze the morphology of modified electrodes, working interface was manually cut and analyzed through TESCAN VEGA 3 for SEM micrographs. An (XRD-6000) diffractometer was employed for X-ray diffraction study of synthesized materials at voltage of 40 kV using monochromated Cu Kα radiation (λ = 1.54 Å, 40 kV, 30 mA). Raman (Renishaw invia microscope) was used to calculate ratio of inplane vibrations of the sp2 carbon (G-band) and disorder-induced mode (D-band) at excitation wavelength.

1.2 Instrumentation

For electrochemical analysis Amel-2553 potentiostat/galvanostat equipped with ZPlus software was used. All the experiments were conducted at room temperature with a three-electrode system containing GPE as working, Ag/AgCl/Sat. KCl electrode as a reference electrode with a standard potential of (E=+0.197 V saturated), and platinum based counter electrode. Electrochemical Impedance Spectroscopy (EIS) measurements were performed in the presence of 5 mM ferro/ferri solution (1:1) in the range of 0.1 Hz-100000 Hz. Working interface i.e. graphitic pencil electrode was manually cut and used for microscopic analysis. Fourier Transform Infrared (FTIR) spectrums were recorded on Thermo scientific Nicolet 6700 in ATR mode to examine the functional groups exist in the pristine and composite materials. The X-ray diffraction (XRD) measurements by using a Rigaku D/max-2550 instrument equipped with a Cu-Kα radiation source (λ=1.5418 Å) has been employed to analyze phase composition. Raman spectrums were recorded on Renishaw in Via-reflex spectrometer. The surface morphologies of modified electrodes were studied by scanning electron microscopy (SEM) at TESCAN VEGA 3 and atomic force microscopy (AFM) at Park Systems AFM XE7 in non-contact mode and.

2 Results and Discussion Section

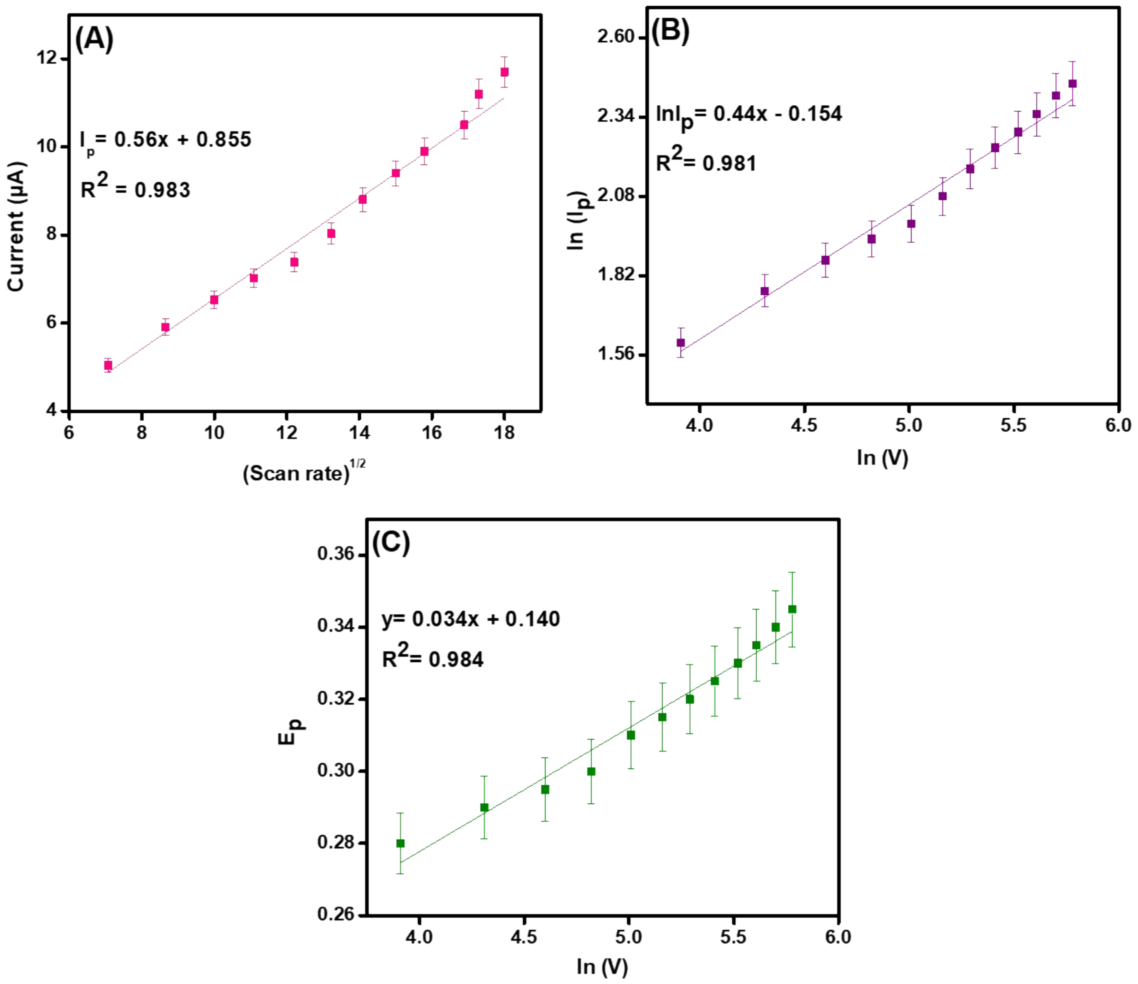

Fig S1. Linear graph of CuO-PDI-GPE showing relationship between square root of scan rate and oxidative peak current (A), linear plot showing relationship between natural log of scan rate and natural log of anodic peak current (B) and linear graph showing relation between natural log of scan rate versus oxidative peak potential (C).

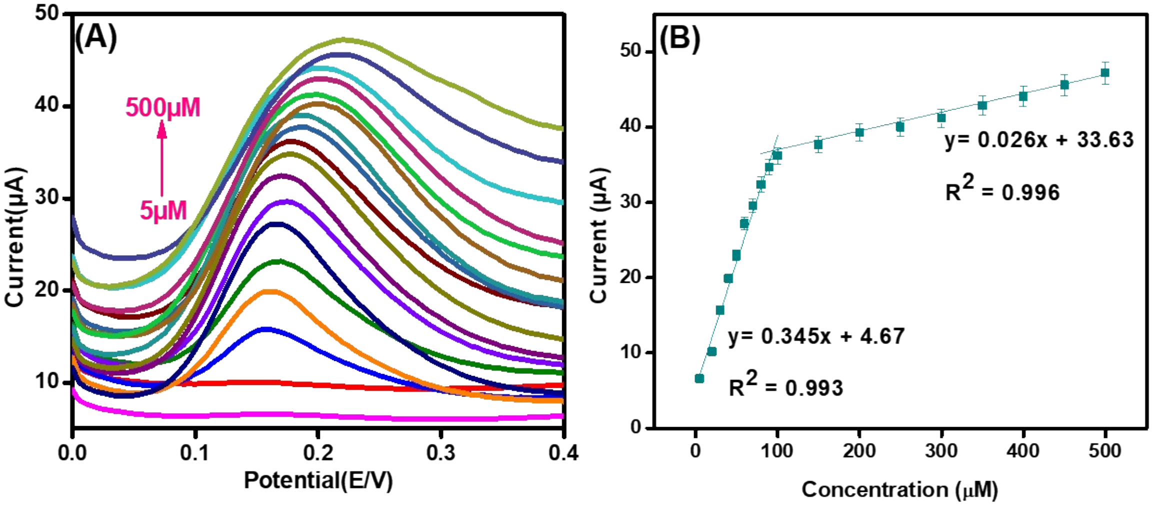

Fig S2. Amperometry (A) and its corresponding linear graph (B) for CuO-PDI-GPE at concentrations bounds of 5 μM to 500 μM in phosphate buffer (pH 7.4) at a scan rate of 50 mV/s.

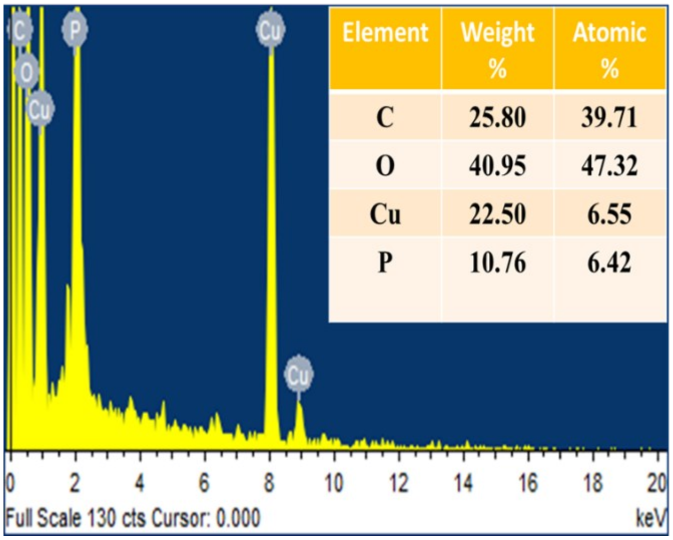

Fig S3. EDX analysis of CuO-PDI-GPE.

Table S1: Comparison of major features of CuO-PDI-GPE and previously reported modified surfaces for dopamine determination.

Electrode matrix	Sensitivity ($\mu A\ \mu M^{-1}\ cm^{-2}$)	LOD (nM)	Linear range (μM)	Reproducibility (RSD %)	Ref.
CuO nanostructures	0.012	110	5-40	>5	[1]
TC-GQD/GCE	4.9	220	1-500	-	[2]
CuO/CN-5	0.331	60	16-78.7	-	[3]
rGO–Cu_2O	10.52	50	10-900	4.2	[4]
MBIP/PGE	-	6	0.02-7	3.5	[5]
Sn@rGO/MnO_2	0.092	120	0–50	-	[6]
(HNP) PtTi alloy	-	3200	4-500	2.5	[7]
N-rGO-180-8/NH_3	1.82	410	0.5–150	6.22	[8]
AuNPs@MIPs	-	7.8	0.02-0.54	4.4	[9]
Mo NPs@*f*-MWCNTs	4.925	1.26	0.01-161	2.8	[10]
N2/ Ar/GS/GNR/GCE	652	2.5	0.01-400	2.2	[11]
S-Fe_2O_3 NPs−Nafion	0.1315	31.25	0.2-107	-	[12]
Au@ZIF-8	0.006	10	0.1-50	0.9-3.3	[13]
Ppy-PBA/GCE	-	33	0.05-10	4.3	[14]
**CuO-PDI-GPE**	**4**	**6**	**5-100**<br>**100-500**	**2.9**	**This work**

TC-GQD/GCE= titania-ceria-graphene quantum dots, CuO/CN-5=copper oxide/carbon nitride, rGO–Cu_2O= opper (I) oxide nanostructure decorated reduced graphene oxide, MBIP= molecularly bioimprinted polymer, Sn= Stannum, MnO_2 = Maganese oxide, rGO= reduced graphene oxide, (HNP) PtTi alloy= hierarchical nanoporous PtTi alloy, N-rGOs= N-doped reduced graphene oxides, AuNPs@MIPs= gold nanoparticles doped molecularly imprinted polymers, Mo= Molebdenum, f-MWCNT= Functionalized carbon nanotubes , N2/ Ar/GS/GNR= nitrogen/argon plasma functionalized graphene nanosheet/graphene nanoribon, S-Fe_2O_3 NPs = shuttle like hematite nanoparticles, Au@ZIF-8=Gold NPs@zeolitic imidazolate, Ppy-PBA= Pyrrole-phenylboronic acid.

References

[1] A. Nafady, A. Tahira, S.T.H. Sherazi, T. Shaikh, M. Arain, M. Willander, et al., An amperometric sensitive dopamine biosensor based on novel copper oxide nanostructures, Microsystem Technologies, 23(2017) 1229-35.

[2] N. Ahmadi, M. Bagherzadeh, A. Nemati, Comparison between electrochemical and photoelectrochemical detection of dopamine based on titania-ceria-graphene quantum dots nanocomposite, Biosensors and Bioelectronics, 151(2020) 111977.

[3] Y. Huang, Y. Tan, C. Feng, S. Wang, H. Wu, G. Zhang, Synthesis of CuO/gC 3 N 4 composites, and their application to voltammetric sensing of glucose and dopamine, Microchimica Acta, 186(2019) 10.

[4] R. Sivasubramanian, P. Biji, Preparation of copper (I) oxide nanohexagon decorated reduced graphene oxide nanocomposite and its application in electrochemical sensing of dopamine, Materials Science and Engineering: B, 210(2016) 10-8.

[5] B. Rezaei, M.K. Boroujeni, A.A. Ensafi, Fabrication of DNA, o-phenylenediamine, and gold nanoparticle bioimprinted polymer electrochemical sensor for the determination of dopamine, Biosensors and Bioelectronics, 66(2015) 490-6.

[6] D. Shanbhag, K. Bindu, A. Aarathy, M. Ramesh, M. Sreejesh, H. Nagaraja, Hydrothermally synthesized reduced graphene oxide and Sn doped manganese dioxide nanocomposites for supercapacitors and dopamine sensors, Materials today energy, 4(2017) 66-74.

[7] D. Zhao, G. Yu, K. Tian, C. Xu, A highly sensitive and stable electrochemical sensor for simultaneous detection towards ascorbic acid, dopamine, and uric acid based on the hierarchical nanoporous PtTi alloy, Biosensors and Bioelectronics, 82(2016) 119-26.

[8] P. Wiench, Z. González, R. Menéndez, B. Grzyb, G. Gryglewicz, Beneficial impact of oxygen on the electrochemical performance of dopamine sensors based on N-doped reduced graphene oxides, Sensors and Actuators B: Chemical, 257(2018) 143-53.

[9] C. Xue, Q. Han, Y. Wang, J. Wu, T. Wen, R. Wang, et al., Amperometric detection of dopamine in human serumby electrochemical sensor based on gold nanoparticles doped molecularly imprinted polymers, Biosensors and Bioelectronics, 49(2013) 199-203.

[10] M. Keerthi, G. Boopathy, S.-M. Chen, T.-W. Chen, B.-S. Lou, A core-shell molybdenum nanoparticles entrapped f-MWCNT s hybrid nanostructured material based non-enzymatic biosensor for electrochemical detection of dopamine neurotransmitter in biological samples, Scientific reports, 9(2019) 1-12.

[11] L. Jothi, S. Neogi, S. kumar Jaganathan, G. Nageswaran, Simultaneous determination of ascorbic acid, dopamine and uric acid by a novel electrochemical sensor based on N2/Ar RF plasma assisted graphene nanosheets/graphene nanoribbons, Biosensors and Bioelectronics, 105(2018) 236-42.

[12] A. Chen, L. Xu, X. Zhang, Z. Yang, S. Yang, Improving surface adsorption via shape control of hematite α-Fe2O3 nanoparticles for sensitive dopamine sensors, ACS applied materials & interfaces, 8(2016) 33765-74.

[13] L.F. de Lima, C.M. Daikuzono, C.M. Miyazaki, E.A. Pereira, M. Ferreira, Layer-by-Layer nanostructured films of magnetite nanoparticles and polypyrrole towards synergistic effect on methylparaben electrochemical detection, Applied Surface Science, 505(2020) 144278.

[14] M. Zhong, Y. Teng, S. Pang, L. Yan, X. Kan, Pyrrole–phenylboronic acid: A novel monomer for dopamine recognition and detection based on imprinted electrochemical sensor, Biosensors and Bioelectronics, 64(2015) 212-8.
